# Evaluation of the Diagnostic Efficacy of Xpert CT/NG for *Chlamydia trachomatis* and *Neisseria gonorrhoeae*

**DOI:** 10.1155/2020/2892734

**Published:** 2020-10-08

**Authors:** Tian-Ao Xie, Ye-Ling Liu, Rui-Chun Meng, Xiao-Shan Liu, Ke-Ying Fang, Shu-Ting Deng, Shu-Jin Fan, Chu-Mao Chen, Qin-Rong Lin, Zhi-Jian He, Zhen-Xing Li, Shi Ouyang, Guo-Dong Zhu, Tian-Xing Ji, Yong Xia, Zhi-Yong Pan, Xu-Guang Guo

**Affiliations:** ^1^Department of Clinical Medicine, The Third Clinical School of Guangzhou Medical University, Guangzhou 511436, China; ^2^Department of Clinical Laboratory Medicine, The Third Affiliated Hospital of Guangzhou Medical University, Guangzhou 510150, China; ^3^Department of Respiratory Medicine, The Third Affiliated Hospital of Guangzhou Medical University, Guangzhou 510150, China; ^4^Department of Infectious Disease, The Fifth Affiliated Hospital of Guangzhou Medical University, 510000, China; ^5^The Second Affiliated Hospital of South China University of Technology, Geriatrics Related Fundamental and Clinical Research, 510180, China; ^6^Department of Clinical Medicine, The Second Affiliated Hospital of Guangzhou Medical University, 511436, China; ^7^Key Laboratory for Major Obstetric Diseases of Guangdong Province, The Third Affiliated Hospital of Guangzhou Medical University, Guangzhou 510150, China; ^8^Key Laboratory of Reproduction and Genetics of Guangdong Higher Education Institutes, The Third Affiliated Hospital of Guangzhou Medical University, Guangzhou 510150, China

## Abstract

**Background:**

*Chlamydia trachomatis* (CT) and *Neisseria gonorrhoeae* (NG) are widely spread across the world. Asymptomatic or inconspicuous CT/NG infections are difficult to diagnose and treat. Traditional methods have the disadvantages of low detection rate, inaccurate results, and long detection time. However, Xpert CT/NG makes up for the aforementioned shortcomings and has research value and popularization significance.

**Methods:**

PubMed, Embase, Cochrane Library, and Web of Science were systematically searched, and studies were screened using Xpert CT/NG for diagnosing CT/NG. QUADAS-2 was used to evaluate the quality of the eligible studies. Then, two groups of researchers independently extracted data from these studies. Meta-analyses of sensitivity (SEN), specificity (SPE), positive likelihood ratio (PLR), negative likelihood ratio (NLR), diagnostic odds ratio (DOR), and the area under the curve (AUC) of the summary receiver operating characteristic (SROC) curve were conducted using Meta-DiSc 1.4. Finally, Deek's funnel plots were made using Stata 12.0 to evaluate publication bias.

**Results:**

14 studies were identified, and 46 fourfold tables were extracted in this meta-analysis. The pooled SEN, SPE, PLR, NLR, DOR, and AUC in diagnosing CT were 0.94 (95% confidence interval (CI): 0.93–0.95), 0.99 (95% CI: 0.99–1.00), 97.17 (95% CI: 56.76–166.32), 0.07 (95% CI: 0.04–0.12), 1857.25 (95% CI: 943.78–3654.86), and 0.9960, respectively. The pooled SEN, SPE, PLR, NLR, DOR, and AUC in diagnosing NG were 0.95 (95% CI: 0.93–0.96), 1.00 (95% CI: 1.00–1.00), 278.15 (95% CI: 152.41–507.63), 0.08 (95% CI: 0.06–0.12), 4290.70 (95% CI: 2161.78–8516.16), and 0.9980, respectively.

**Conclusions:**

Xpert CT/NG had high diagnostic sensitivity and specificity for CT and NG. However, more evidence is required to confirm that Xpert CT/NG might serve as the primary method for detecting CT and NG and even the gold standard for diagnosis in the future.

## 1. Background

*Chlamydia trachomatis* (CT) and *Neisseria gonorrhoeae* (NG) are the most common infectious bacteria in sexually transmitted infections (STIs) [1]. According to incomplete statistics, CT and NG are widely spread across the world (about 131 million and 78 million, respectively) [2]. Their rate of infection has also increased every year, with a larger fraction in women [3].

CT, a prokaryotic microorganism, is divided into 19 serotypes. In these serotypes, serotypes A to C mainly cause trachoma, serotypes D to K cause urogenital tract infection, and serotypes L1 to L3 cause lymphogranuloma venereum [4]. The elementary body of CT is one of the two bacterial morphologies that cannot split but is extremely infected. It can differentiate into reticulate bodies (RBs) around the epithelial cells. RBs infect other cells after replication by two divisions, facilitating the spread of CT [5]. Quite a few people infected with CT are asymptomatic or inconspicuous, especially those with genital tract infection [6]. The failure to seek timely medical care can lead to serious complications, such as male urethritis, epididymitis, female cervicitis, and pelvic inflammatory disease [1, 2]. Children have trachoma more commonly, while newborns acquire CT infection through the genital tracts of their mothers and suffer neonatal ophthalmia and pneumonia [4]. Therefore, the differential diagnosis of CT infection needs immediate attention.

Humans are the natural host of NG [7]. More than one million STIs are acquired every day worldwide [8, 9], and NG mainly causes gonorrhea. When NG intrudes tissues of the body, it uses its pili and outer membrane proteins to adhere to mucosal epithelial cells, leading to invasion with endocytosis. Subsequently, NG reproduces fast in host cells causing cell lysis and then enters the submucosa to produce toxins leading to inflammatory reactions [10]. Hence, the body produces purulent secretions representatively. Moreover, up to 50% to 80% of women and about 10% of men have asymptomatic infections [11]. Antibiotics are frequently prescribed to treat gonorrhea. With this widespread use, NG becomes resistant to antibiotics, including extended-spectrum cephalosporin ceftriaxone, affecting the treatment and prognosis of gonococcal infection [12]. In the meantime, NG develops a mechanism to escape from the human immune system [13]. This leads to a dramatic increase in difficulty of treating NG infection.

CT/NG infections can be diagnosed by the antigen–antibody test, serological examination, and nucleic acid amplification tests (NAATs). The indirect methods of detection depend on antibodies with a low detection rate of pathogens. The serological examination is not suitable for acute response in that it usually takes weeks to months with the low accuracy of test results [14]. The use of NAATs is the preferred diagnostic method with high specificity and sensitivity at present, but it still takes several days to detect bacteria [15].

The GeneXpert (Cepheid, USA) is an automatic molecular diagnosis method based on the nucleic acid amplification test [16]. The core of detection technology is the application of 90 min real-time fluorescent quantitative polymerase chain reaction (FQ-PCR), which has the same performance as traditional NAAT [1]. The Xpert platform integrates and automates sample preparation, nucleic acid purification, gene amplification, and result reporting in quantitative PCR molecular detection [17]. CT and NG pathogens can be detected rapidly on the same day at the same time. The advantages of the Xpert system include the ability to use each machine module independently, allowing the simultaneous screening of multiple samples. It is faster, simpler, more accurate, and safer than the traditional methods [18].

The Xpert CT/NG kit is one type of Xpert platform [16]. Its samples mainly come from the female cervical endometrial swab, vaginal swab, and female or male urine. Subsequently, DNA of CT and NG can be detected using the kit-based assay [16, 19]. Currently, no meta-analysis has investigated the diagnostic accuracy of GeneXpert for CT and NG in the field of evidence-based medicine. This meta-analysis is aimed at exploring the sensitivity and specificity of Cepheid Xpert CT/NG in the diagnosis of CT and NG infections.

## 2. Methods

### 2.1. Study Design

This study was conducted from March 2019 to date. The sensitivity and specificity of Xpert CT/NG in identifying CT and NG were systematically evaluated.

### 2.2. Electronic Searches

PubMed, Embase, the Cochrane Library, and Web of Science were systematically searched using the following terms: (Xpert CT/NG OR Xpert OR GeneXpert OR Xpert CT OR Xpert NG OR Cepheid) AND (*Chlamydia trachomatis* [all synonyms] OR *Neisseria gonorrhoeae* [all synonyms]) (last search: 5 March 2020). There are language restrictions: we only include English literature. Relevant studies from January 2000 to March 2020 from the aforementioned four databases were included.

### 2.3. Inclusion and Exclusion Criteria

The studies were searched from the database, and the details were imported into EndNote X8. The four members of the research team were divided into two groups (TA Xie and RC Meng; YL Liu and XS Liu). Each group was responsible for half of the studies. Duplicate documents were independently examined and deleted. Also, the literature abstracts were reviewed, and the full text was read carefully. In the case of a problem that could not be solved within the group, another person was selected as a third party to negotiate and solve it. The inclusion and exclusion criteria were established for suitability of studies, and all search results were evaluated. The inclusion criteria were as follows: (1) the research type must study the clinical application effect of Xpert CT/NG kit, (2) the target should be the carrier of CT/NG, (3) the diagnostic method should use the Xpert CT/NG kit, and (4) the measurement index should also be the content of CT/NG. The exclusion criteria were as follows: (1) the conference abstract was excluded because quality evaluation could not be conducted, (2) studies in which fourfold tables could not be extracted were excluded, and (3) letters and reviews were excluded because they were incomplete and difficult to meta-analysis.

### 2.4. Data Extraction

According to the previous inclusion and exclusion criteria, conference abstracts, letters, reviews, and studies in which fourfold tables could not be extracted were excluded. The remaining studies that met the inclusion criteria were retained, and the data of the included articles were extracted independently by the two groups. The extracted data included author, year, country, study design, gold standard, source of specimens, bacterial species, type of specimens, and result.

### 2.5. Assessment of Quality of Studies

After data extraction, the quality of the study included in the meta-analysis was evaluated by referring to the Cochrane collaboration QUADAS-2 standard, in which the responses “Yes,” “Unclear,” and “No” were used for evaluation [20].

### 2.6. Data Analysis

Meta-DiSc 1.4 was used to integrate the sensitivity and specificity, fit SROC curve, and provide common likelihood ratio, diagnostic odds ratio (DOR), heterogeneity test, and meta-regression analysis functions for the sensitivity, specificity, positive LR, negative LR, diagnostic OR, and SROC curve map [21]. Then, Deek's funnel plots were made using Stata 12.0 to evaluate publication bias.

## 3. Results

### 3.1. Search Results

Based on the previous retrieval strategy, 326 relevant studies were obtained, including 76 in PubMed, 153 in Embase, 12 in Cochrane, and 85 in the Web of Science. After deletion of duplicate studies, 185 studies remained. Then, 90 irrelevant studies were excluded after reviewing the abstract. After full-text review, finally, fourteen studies [22–35] were included in the meta-analysis. Among the excluded studies, 81 were excluded because they did not meet the inclusion criteria, including 2 reviews, 3 letters, 1 note, 52 conference abstracts, 1 clinical trial protocol, and 22 articles in which fourfold tables could not be extracted. From the remaining 14 studies, 46 sets of research data were extracted (Supplementary Figure 1). In total, we extracted 13442 specimens from the studies.

### 3.2. Characteristics of Studies

Information on author, year, country, study design, gold standard, source of specimens, bacterial species, type of specimens, and result was extracted from the included eight studies. The characteristics of the studies on CT are summarized in Table 1. The characteristics of the studies on NG are summarized in Table 2.

### 3.3. Quality Assessment

The quality of these eight studies was evaluated using the QUADAS-2. In the process of evaluation, “Yes,” “Unclear,” and “No” responses were used to assess the studies. The specific quality assessment results are shown in Table 3.

### 3.4. Merge Analysis Results

Meta-DiSc 1.4 software was used to analyze the fourfold table data extracted from these eight studies.

**Figure 1:**
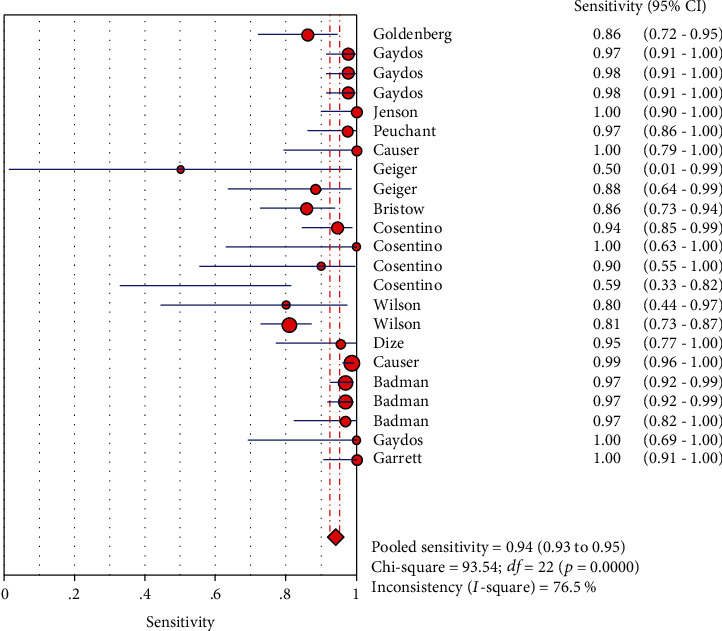
Forest plots of the combined sensitivity of Xpert CT/NG for the diagnosis of *Chlamydia trachomatis*.

**Figure 2:**
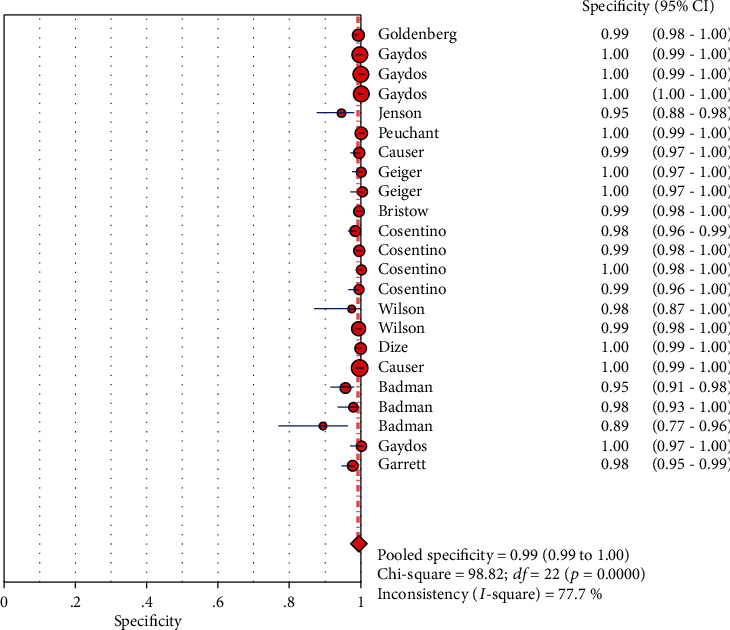
Forest plots of the combined specificity of Xpert CT/NG for the diagnosis of *Chlamydia trachomatis*.

**Figure 3:**
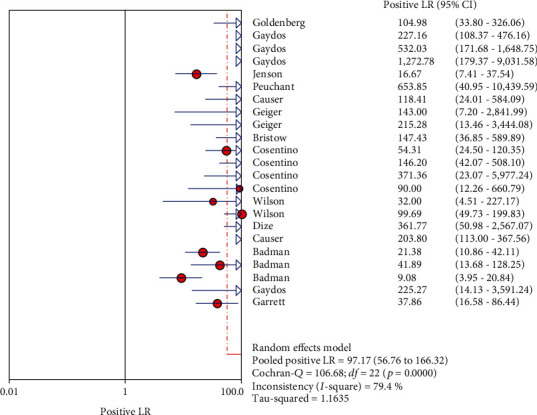
Forest plots of the combined positive LR of Xpert CT/NG for the diagnosis of *Chlamydia trachomatis*.

**Figure 4:**
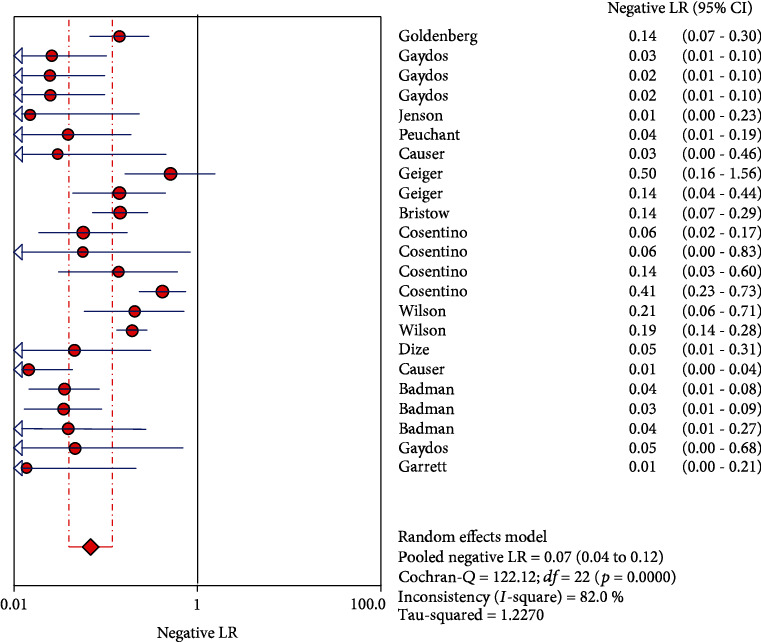
Forest plots of the combined negative LR of Xpert CT/NG for the diagnosis of *Chlamydia trachomatis*.

**Figure 5:**
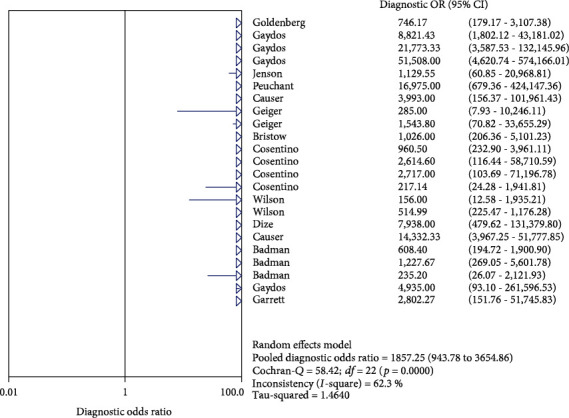
Forest plots of the combined diagnostic OR of Xpert CT/NG for the diagnosis of *Chlamydia trachomatis*.

The results on CT are shown in Figures 1–5. Xpert CT/NG was used to detect CT merger sensitivity, specificity, positive LR, negative LR, and diagnostic OR; the values were 0.94 (95% CI: 0.93–0.95), 0.99 (95% CI: 0.99–1.00), 97.17 (95% CI: 56.76–166.32), 0.07 (95% CI: 0.04–0.12), and 1857.25 (95% CI: 943.78–3654.86), respectively.

**Figure 6:**
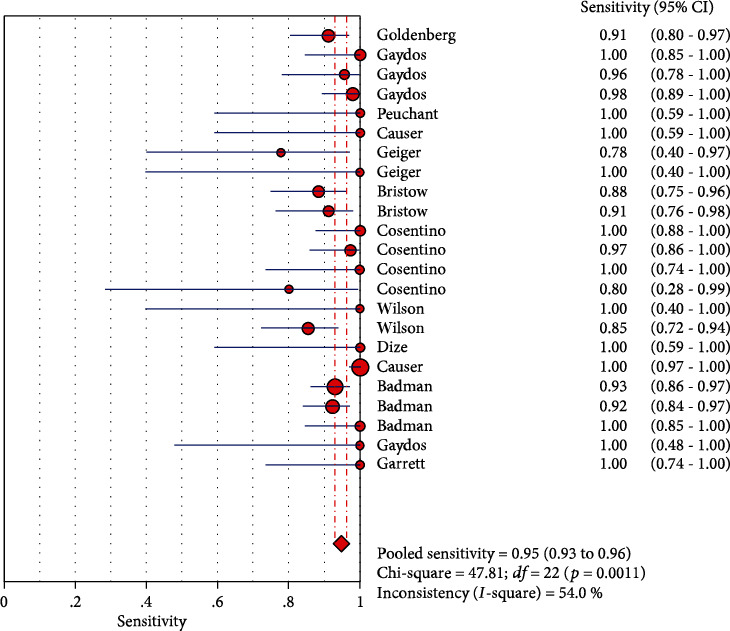
Forest plots of the combined sensitivity of Xpert CT/NG for the diagnosis of *Neisseria gonorrhoeae*.

**Figure 7:**
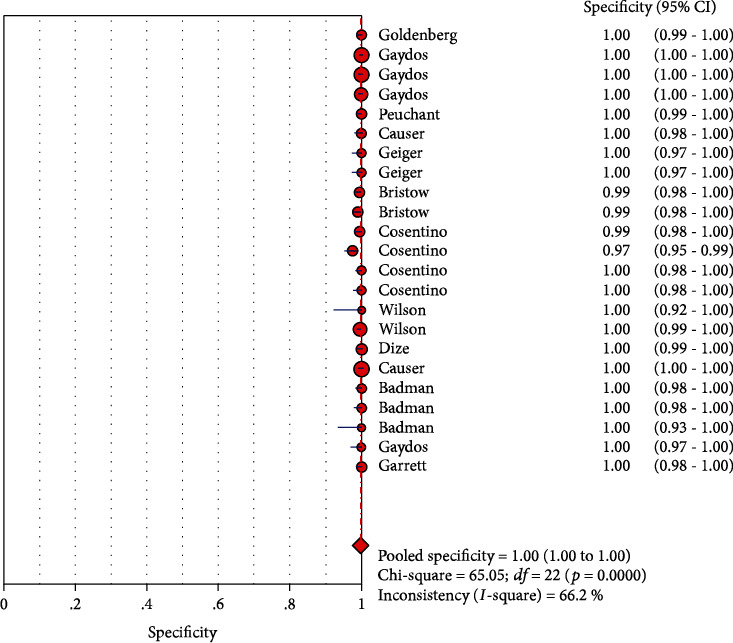
Forest plots of the combined specificity of Xpert CT/NG for the diagnosis of *Neisseria gonorrhoeae*.

**Figure 8:**
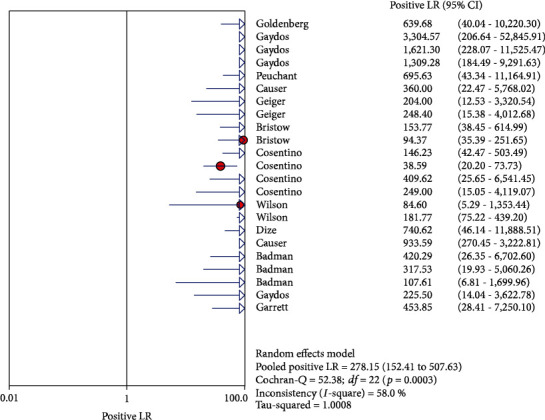
Forest plots of the combined positive LR of Xpert CT/NG for the diagnosis of *Neisseria gonorrhoeae*.

**Figure 9:**
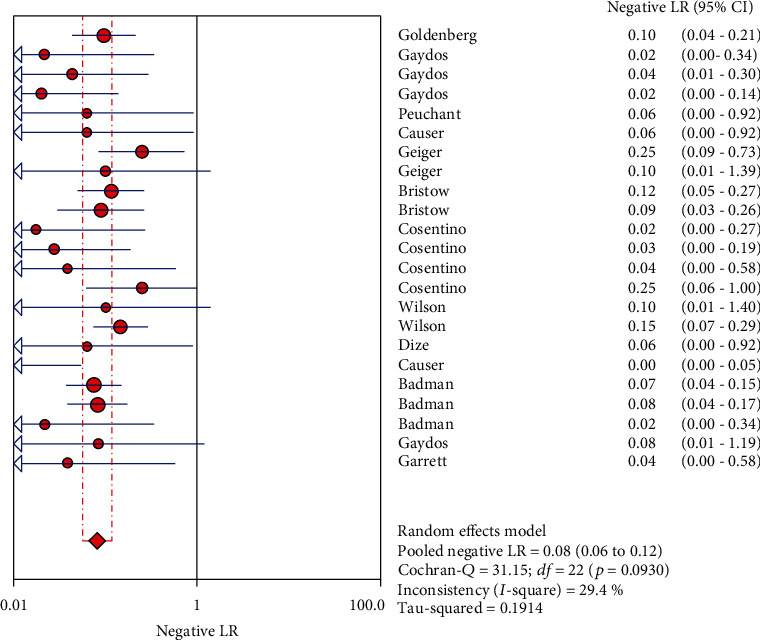
Forest plots of the combined negative LR of Xpert CT/NG for the diagnosis of *Neisseria gonorrhoeae*.

**Figure 10:**
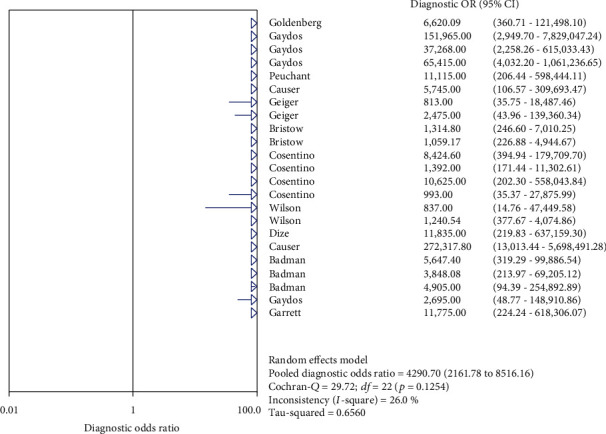
Forest plots of the combined diagnostic OR of Xpert CT/NG for the diagnosis of *Neisseria gonorrhoeae*.

The results on NG are shown in Figures 6–10. Xpert CT/NG was used to detect CT merger sensitivity, specificity, positive LR, negative LR, and diagnostic OR; the values were 0.95 (95% CI: 0.93–0.96), 1.00 (95% CI: 1.00–1.00), 278.15 (95% CI: 152.41–507.63), 0.08 (95% CI: 0.06– 0.12), and 4290.70 (95% CI: 2161.78–8516.16), respectively.

### 3.5. SROC Curve

**Figure 11:**
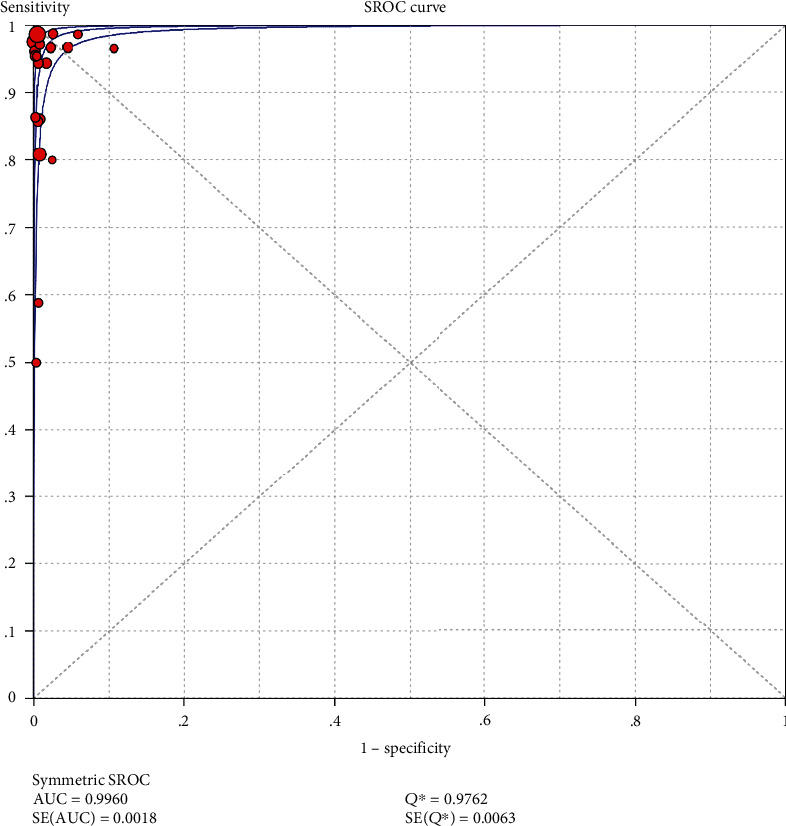
The summary receiver operating characteristic (SROC) curve of Xpert CT/NG in *Chlamydia trachomatis*.

**Figure 12:**
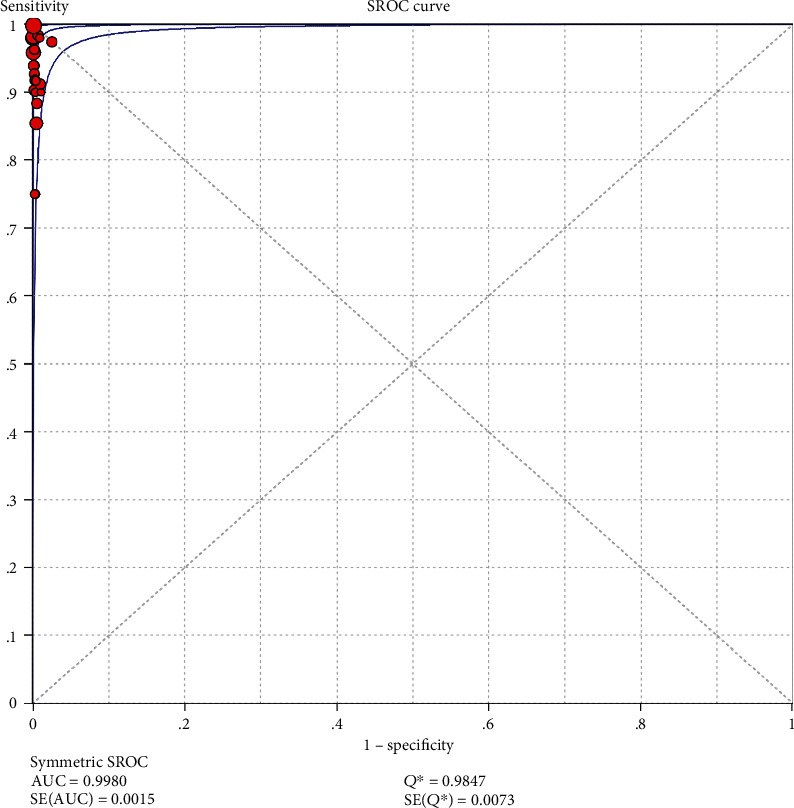
The summary receiver operating characteristic (SROC) curve of Xpert CT/NG in *Neisseria gonorrhoeae*.

The SROC curve on CT is shown in Figure 11 (AUC = 0.9960; *Q* index 0.9762; SE = 0.0063). The SROC curve on NG is shown in Figure 12 (AUC = 0.9980; *Q* index 0.9847; SE = 0.0073). As the AUC values of both were very close to 1, it was suggested that Xpert CT/NG had high identification accuracy for CT and NG.

### 3.6. Subgroup Analysis

Significant heterogeneity was found in these studies because *I*-square values were more than 50% (Figures 1, 2, 6, and 7).

In the subgroup analysis of specimen type in CT, the pooled sensitivity of anorectal, urine, and vaginal specimens was 0.94 (95% CI: 0.91–0.96; *I*^2^: 47.1%), 0.90 (95% CI: 0.87–0.93; *I*^2^: 82.2%), and 0.91 (95% CI: 0.84–0.96; *I*^2^: 90.9%), respectively. The pooled specificity of anorectal, urine, and vaginal specimens was 0.99 (95% CI: 0.98–0.99; *I*^2^: 78.6%), 1.00 (95% CI: 0.99–1.00; *I*^2^: 63.9%), and 0.99 (95% CI: 0.98–1.00; *I*^2^: 79.6%), respectively.

In the subgroup analysis of specimen type in NG, the pooled sensitivity of anorectal, urine, and vaginal specimens was 0.93 (95% CI: 0.90–0.96; *I*^2^: 32.6%), 0.94 (95% CI: 0.88–0.97; *I*^2^: 46.9%), and 0.96 (95% CI: 0.79–1.00; *I*^2^: 39.6%), respectively. The pooled specificity of anorectal, urine, and vaginal specimens was 1.00 (95% CI: 1.00–1.00; *I*^2^: 16.9%), 1.00 (95% CI: 1.00–1.00; *I*^2^: 35.9%), and 1.00 (95% CI: 1.00–1.00; *I*^2^: 0.0%), respectively (Table 4).

In the subgroup analysis of gender in CT, the pooled sensitivity of female and male individuals was 0.91 (95% CI: 0.87–0.93; *I*^2^: 87.4%) and 0.93 (95% CI: 0.87–0.97; *I*^2^: 52.6%), respectively. The pooled specificity of female and male individuals was 1.00 (95% CI: 0.99–1.00; *I*^2^: 67.5%) and 1.00 (95% CI: 1.00–1.00; *I*^2^: 28.1%), respectively.

In the subgroup analysis of gender in NG, the pooled sensitivity of female and male individuals was 0.93 (95% CI: 0.86–0.97; *I*^2^: 46.1%) and 0.96 (95% CI: 0.89–0.99; *I*^2^: 35.2%), respectively. The pooled specificity of female and male individuals was 1.00 (95% CI: 1.00–1.00; *I*^2^: 54.0%) and 1.00 (95% CI: 1.00–1.00; *I*^2^: 0.0%), respectively (Table 5).

### 3.7. Publication Bias

**Figure 13:**
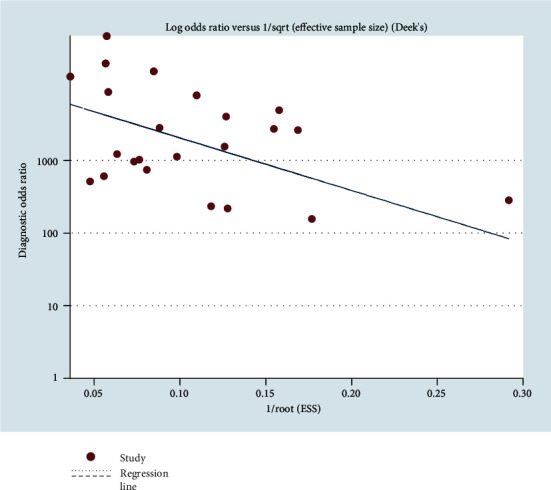
Deek's funnel plot asymmetry test to assess publication bias for Xpert CT/NG detection of *Chlamydia trachomatis*.

**Figure 14:**
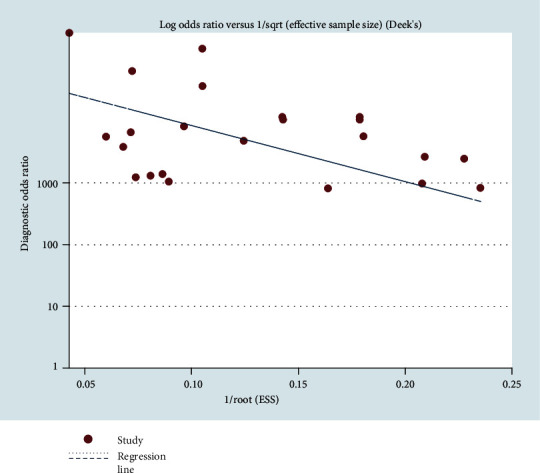
Deek's funnel plot asymmetry test to assess publication bias for Xpert CT/NG detection of *Neisseria gonorrhoeae*.

The potential publication bias of these eight studies was evaluated using Deek's funnel plot asymmetry test. No publication bias was found in Deek's funnel plot of CT (Figure 13). The Egger test indicated that the publication bias of these studies in CT was low (*p* = 0.122). No publication bias was found in Deek's funnel plot of NG (Figure 14). The Egger test indicated that the publication bias of these studies in NG was low (*p* = 0.048).

## 4. Discussion

CT and NG infections have become a major threat to human health [3]. In this context, the diagnostic efficacy of Xpert CT/NG for CT and NG becomes particularly important. In this meta-analysis, eight studies were searched and screened. A scientific and systematic evaluation of the diagnostic efficacy of Xpert CT/NG was conducted by analyzing the data extracted from the eight studies.

In this study, the results of systematic evaluation revealed the following: (1) In CT, the sensitivity, specificity, positive likelihood ratio (PLR); negative likelihood ratio (NLR); and DOR of Xpert CT/NG were 0.94, 0.99, 97.17, 0.07, and 1857.25, respectively. (2) In NG, the sensitivity, specificity, PLR, NLR, and DOR of Xpert CT/NG were 0.95, 1.00, 278.15, 0.08, and 4290.70, respectively. The positive LRs of CT and NG were both much larger than 10, and the negative LRs were both less than 0.1. The SROC AUC of CT and NG was 0.9956 and 0.9980, respectively; both of which were very close to 1. Besides, the SROC curves of both were very close to the upper left corner. Thus, it indicated that the diagnostic accuracy of Xpert CT/NG for CT and NG was very high. According to the results of the funnel plots of CT and NG, despite some asymmetric phenomena in the graphs of both due to the limited number of studies, the *p* values (0.122 and 0.048) were greater than 0.01, with no statistical significance, indicating that the possibility of publication bias was low.

Due to the obvious heterogeneity in this study, the subgroup analysis of CT and NG was conducted based on the sample type and gender to facilitate further description of the results. The results of CT analysis in sample type showed that *I*^2^ values were more than 50% except for the combined sensitivity in “anorectal,” which was 47.1%, indicating high heterogeneity. In terms of gender, *I*^2^ values were higher than 50% for both “male” and “female,” but they were obviously higher for “female” than for “male.” Therefore, heterogeneity existed in both “male” and “female,” and “female” was higher in comparison. Meanwhile, the result of NG analysis in sample type showed that *I*^2^ values were lower than 50%, indicating low heterogeneity. In terms of gender, *I*^2^ values of specificity in “female” were slightly higher than 50%, while *I*^2^ values of “male” were lower than 50%, indicating low heterogeneity in “female,” but not in “male.” After in-depth study of these fourteen studies, it was believed that the reasons for the heterogeneity might be the following: (1) the specimens collected were either degraded or poorly mixed in the study [24], (2) the improper behavior of the patients when collecting the sample resulted in the incomplete specimens [28], and (3) before collecting vaginal specimens, female patients underwent vaginal cleansing [25]. The sensitivity and specificity of CT and NG in sample type were both greater than 0.9, and the specificity of NG was 1. In terms of sex, the combined sensitivity of CT and NG was greater than 0.9, and the specificity was 1. These results, on the one hand, indicated that Xpert CT/NG had a high accuracy in identifying anorectal, urethral, and vaginal specimens. On the other hand, no obvious sex-related difference existed in the identification accuracy of Xpert CT/NG for CT and NG. In addition, the data of “endocervical,” “ocular,” and “pharyngeal” were also extracted, but the subgroup analysis could not be conducted because of insufficient data. After the direct analysis of the data, it was not difficult to find that Xpert CT/NG also had high sensitivity and specificity in the identification of endocervical, ocular, and pharyngeal samples.

Most of the NAATs use the fluorescence probe technology to detect amplified products in real time based on PCR. Compared with culture, conventional NAATs do not rely on live bacteria when detecting CT and NG and therefore are very beneficial for sample transportation [14]. With the same sensitivity and specificity as culture, the conventional NAATs have replaced culture as the gold standard [36]. In 2002, the Centers for Disease Control and Prevention (CDC) recommended that NAAT-positive results should be confirmed with a second test to prevent unnecessary treatment and psychological testing. However, subsequent studies showed that unconfirmed positive results were not necessarily false positive, but may also be false negative, indirectly leading to the uncertainty of NAAT test results [37]. Sometimes, when the concentrations of some samples were too low, the NAAT detection rate was also very low [38]. In addition, some variants such as the Swedish variant (*C. trachomatis* strain E/SW2) could not be detected due to the defect in the target area of some conventional NAATs [39]. In this case, Xpert CT/NG, as an emerging NAAT, has advantages such as unique integration, allowing the simultaneous detection of multiple samples without interference [18]. Compared with the conventional NAATs, Xpert CT/NG has eliminated the existing shortcomings and improved the detection accuracy of CT and NG. The most striking thing is that the speed of detecting CT and NG has greatly increased [1], facilitating the timely treatment of patients. Therefore, Xpert CT/NG might become the preferred method for detecting CT and NG in the future.

In addition, Bristow et al. [40] published a new meta-analysis similar to our study in 2019. We carefully read and studied this article, and compared our study with it, and found several differences: (1) Compared with the Bristow's study, we searched the PubMed database, as well as the Embase, Cochrane Library, and Web of Science databases, broadening the data source. (2) In their study, only nongenital tract samples of the rectum and pharynx were included. On this basis, we also included ocular samples and genital tract samples like urine and vagina. In addition to analyzing all the data, we also performed a subgroup analysis based on sample type and gender. (3) On the basis of the five literatures included in their study, a total of 14 literatures were included by us. In addition, we found that the heterogeneity of the final results of their study is relatively low compared with ours, but we believe that this is caused by the large and extensive data volume. Therefore, we believe that our study can provide more information for clinical reference.

This study had some limitations. First, despite including all eligible studies in strict accordance with the criteria, it was difficult to ensure that no article was missing. Second, high heterogeneity was found in the analysis of the results, reducing the reliability of the results of this study. Third, only studies in English were searched and included, leading to bias.

## 5. Conclusions

In summary, this meta-analysis showed that Xpert CT/NG had high diagnostic accuracy for CT and NG. Further, Xpert CT/NG had high sensitivity and specificity in the detection of anorectal, urethral, and vaginal samples, with no obvious sex-related difference. Therefore, Xpert CT/NG might become the primary method for detecting CT and NG and even the gold standard for diagnosis in the future. However, the findings need further validation.

## Figures and Tables

**Table 1:** Characteristics about CT from included articles.

Author	Year	Country	Study design	Gold standard	Source of specimens	Bacterial species	Type of specimens	Result			
								TP	FP	FN	TN
Goldenberg	2012	Britain	Prospective	PCR	409 self-collected specimen	CT	Anorectal	37	3	6	363
Gaydos	2013	America	Prospective	PCR	1710 clinical specimens	CT	Endocervical	76	7	2	1625
					1718 clinical specimens		Urine^a^	80	3	2	1633
					1386 clinical specimens		Urine^b^	79	1	2	1304
Jenson	2013	East Africa	Prospective	PCR	144 specimens from trachoma-endemic community	CT	Ocular	35	5	0	87
Causer	2015	Australia	PCR	PCR	198 clinical specimens	CT	Urine	16	1	0	181
Peuchan	2015	France	Prospective	PCR	377 clinical specimens	CT	Vaginal	37	0	1	339
Geiger	2016	America	Prospective	PCR	285 self-collected specimens	CT	Ocular	1	0	1	142
							Anorectal	15	0	2	124
Bristow	2017	America	Prospective	PCR	393 clinical specimens	CT	Anorectal	42	2	7	342
Cosentino	2017	America	Prospective	PCR	399 clinical specimens	CT	Anorectal	51	6	3	339
					394 clinical specimens		Pharyngeal	8	2	0	384
					224 clinical specimens		Urine^b^	9	0	1	214
					170 clinical specimens		Vaginal	10	0	1	152
Dize	2017	America	Prospective	PCR	448 self-collected specimens	CT	Anorectal	21	1	1	378
Wilson	2017	America	Prospective	PCR	50 clinical specimens	CT	Urine^b^	8	1	2	39
					1112 clinical specimens		Urine^a^	101	8	24	979
Causer	2018	Australia	Prospective	PCR	2486 self-collected specimens	CT	Urine, vaginal	209	11	3	2263
Badman	2018	Australia	Prospective	PCR	326 self-collected specimens	CT	Anorectal^c^	144	8	5	169
							Anorectal^d^	116	3	4	127
							Anorectal^e^	28	5	1	42
Gaydos	2018	America	Prospective	PCR	127 clinical specimens	CT	Endocervical	10	0	0	117
Garrett	2019	South Africa	Prospective	PCR	247 clinical specimens	CT	Vaginal	37	5	0	205

^a^These specimens were collected from female. ^b^These specimens were collected from male. ^c^These specimens were collected from “men who have sex with men,” “transgender women,” and “female sex workers.” ^d^These specimens were collected from “female sex workers.” ^e^These specimens were collected from “men who have sex with men” and “transgender women.” TP: true positive; FP: false positive; FN: false negative; TN: true negative.

**Table 2:** Characteristics about NG from included articles.

Author	Year	Country	Study design	Gold standard	Source of specimens	Bacterial species	Type of specimens	Result			
								TP	FP	FN	TN
Goldenberg	2012	Britain	Prospective	PCR	409 self-collected specimens	NG	Anorectal	51	0	5	353
Gaydos	2013	America	Prospective	PCR	1710 clinical specimens	NG	Endocervical	22	0	0	1688
					1718 clinical specimens		Urine^a^	22	1	1	1694
					1386 clinical specimens		Urine^b^	49	1	1	1335
Causer	2015	Australia	Prospective	PCR	198 clinical specimens	NG	Urine	7	0	0	191
Peuchan	2015	France	Prospective	PCR	377 clinical specimens	NG	Vaginal	7	0	0	370
Geiger	2016	America	Prospective	PCR	285 self-collected specimens	NG	Ocular	7	0	2	135
							Anorectal	4	0	0	137
Bristow	2017	America	Prospective	PCR	391 clinical specimens	NG	Anorectal	38	2	5	346
					448 clinical specimens		Pharyngeal	31	4	3	410
Cosentino	2017	America	Prospective	PCR	399 clinical specimens	NG	Anorectal	28	2	0	369
					394 clinical specimens		Pharyngeal	36	9	1	348
					224 clinical specimens		Urine^b^	12	0	0	212
					170 clinical specimens		Vaginal	4	0	1	165
Dize	2017	America	Prospective	PCR	448 self-collected specimens	NG	Anorectal	7	0	0	394
Wilson	2017	America	Prospective	PCR	50 clinical specimens	NG	Urine^b^	4	0	0	46
					1112 clinical specimens		Urine^a^	41	5	7	1059
Causer	2018	Australia	Prospective	PCR	2486 self-collected specimens	NG	Urine, vaginal	145	2	0	2339
Badman	2018	Australia	Prospective	PCR	326 self-collected specimens	NG	Anorectal^c^	93	0	7	226
							Anorectal^d^	72	0	6	172
							Anorectal^e^	22	0	0	54
Gaydos	2018	America	Prospective	PCR	127 clinical specimens	NG	Endocervical	5	0	0	122
Garrett	2019	South Africa	Prospective	PCR	247 clinical specimens	NG	Vaginal	12	0	0	235

^a^These specimens were collected from female. ^b^These specimens were collected from male. ^c^These specimens were collected from “men who have sex with men,” “transgender women,” and “female sex workers.” ^d^These specimens were collected from “female sex workers.” ^e^These specimens were collected from “men who have sex with men” and “transgender women.” TP: true positive; FP: false positive; FN: false negative; TN: true negative.

**Table 3:** Quality evaluation of the included articles.

Author	Year	QUADAS-2										
		1	2	3	4	5	6	7	8	9	10	11
Goldenberg	2012	Y	Y	Y	UC	UC	Y	Y	Y	Y	Y	Y
Gaydos	2013	Y	Y	UC	N	UC	Y	Y	UC	Y	Y	N
Jenson	2013	Y	Y	N	Y	UC	Y	Y	N	Y	Y	Y
Causer	2015	Y	Y	Y	Y	UC	Y	UC	Y	Y	Y	Y
Peuchant	2015	Y	Y	Y	Y	UC	Y	N	Y	Y	Y	Y
Geiger	2016	Y	Y	Y	Y	UC	Y	Y	Y	Y	Y	Y
Bristow	2017	Y	Y	UC	N	UC	Y	Y	Y	Y	Y	Y
Cosentino	2017	Y	N	N	Y	UC	Y	N	Y	N	N	Y
Dize	2017	UC	Y	N	N	UC	Y	Y	Y	Y	Y	Y
Wilson	2017	Y	N	N	N	UC	Y	Y	Y	Y	Y	N
Causer	2018	Y	Y	N	Y	UC	Y	N	Y	N	N	Y
Badman	2018	Y	Y	N	Y	UC	Y	N	Y	N	N	N
Gaydos	2018	Y	Y	N	UC	UC	Y	UC	Y	Y	Y	Y
Garrett	2019	Y	Y	N	Y	UC	Y	N	Y	N	N	N

1: Was a consecutive or random sample of patients enrolled?; 2: Was a case-control design avoided.; 3: Did the study avoid inappropriate exclusions?; 4: Were the index test results interpreted without knowledge of the results of the reference standard?; 5: If a threshold was used, was it pre-specified?; 6: Is the reference standards likely to correctly classify the target condition?; 7: Were the reference standard results interpreted without knowledge of the results of the index tests?; 8: Was there an appropriate interval between index test and reference standard?; 9: Did all patients receive the reference standard?; 10: Did all patients receive the same reference standard?; 11: Were all patients included in the analysis?

**Table 4:** Subgroup analyses of Xpert CT/NG for the diagnosis of CT and NG infections in anorectal, urine, and vaginal specimens.

Bacterial species	Type of specimens	Parameter	Estimates	95% CI	*p* value	*I* ^2^
CT	Anorectal	Sensitivity	0.94	0.91-0.96	0.0664	47.1%
		Specificity	0.99	0.98-0.99	0.0000	78.6%
	Urine	Sensitivity	0.90	0.87-0.93	0.0000	82.2%
		Specificity	1.00	0.99-1.00	0.0166	63.9%
	Vaginal	Sensitivity	0.91	0.84-0.96	0.0000	90.9%
		Specificity	0.99	0.98-1.00	0.0075	79.6%
NG	Anorectal	Sensitivity	0.93	0.90-0.96	0.1680	32.6%
		Specificity	1.00	1.00-1.00	0.2970	16.9%
	Urine	Sensitivity	0.94	0.88-0.97	0.0934	46.9%
		Specificity	1.00	1.00-1.00	0.1647	35.9%
	Vaginal	Sensitivity	0.96	0.79-1.00	0.1911	39.6%
		Specificity	1.00	1.00-1.00	1.0000	0.0%

**Table 5:** Subgroup analyses of Xpert CT/NG for the diagnosis of CT and NG infections in gender.

Bacterial species	Gender	Parameter	Estimates	95% CI	*p* value	*I* ^2^
CT	Female	Sensitivity	0.91	0.87-0.93	0.0000	87.4%
		Specificity	1.00	0.99-1.00	0.0051	67.5%
	Male	Sensitivity	0.93	0.87-0.97	0.0770	52.6%
		Specificity	1.00	1.00-1.00	0.2343	28.1%
NG	Female	Sensitivity	0.93	0.86-0.97	0.0844	46.1%
		Specificity	1.00	1.00-1.00	0.0425	54.0%
	Male	Sensitivity	0.96	0.89-0.99	0.1867	35.2%
		Specificity	1.00	1.00-1.00	0.9552	0.0%

## Data Availability

The original data included in our study were from 14 original research articles; all of which can be searched on PubMed database (https://www.ncbi.nlm.nih.gov/pubmed/).

## Abbreviations

CT: Chlamydia trachomatis
NG: Neisseria gonorrhoeae
QUADAS: Quality assessment of diagnostic accuracy studies
SEN: Sensitivity
SPE: Specificity
PLR: Positive likelihood ratio
NLR: Negative likelihood ratio
DOR: Diagnostic odds ratio
AUC: The area under the curve
SROC: The summary receiver operating characteristic
CI: Confidence interval
STIs: Sexually transmitted infections
RBs: Reticulate bodies
NAATs: Nucleic acid amplification tests
FQ-PCR: Fluorescent quantitative polymerase chain reaction
CDC: The Centers for Disease Control and Prevention
PCR: Polymerase chain reaction
TP: True positive
FP: False positive
TN: True negative
FN: False negative
Y: Yes
UC: Unclear
N: No
*I*^2^: Inconsistency (*I*-square).

## Supplementary Materials

Supplementary Materials Supplementary Figure 1: PRISM flow chart for article search to the system.
