# Supplementary figures and images for: Evaluation of the Diagnostic Efficacy of Xpert CT/NG for Chlamydia trachomatis and Neisseria gonorrhoeae
Source: Biomed Res Int. 2020 Oct 8;2020:2892734. doi: 10.1155/2020/2892734 (PMC7576347; doi:10.1155/2020/2892734)

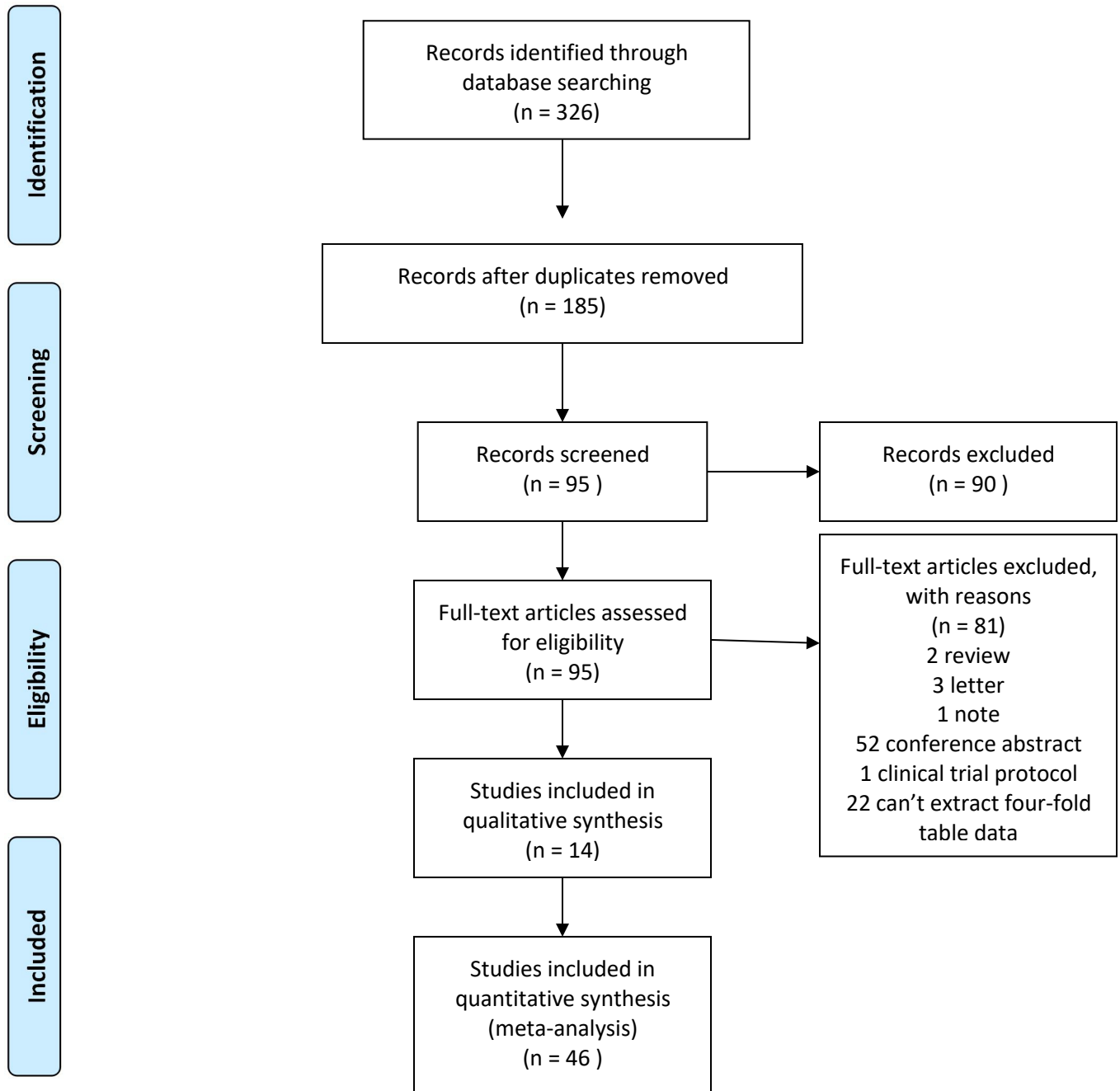

Supplement: Supplementary Materials — Supplementary Figure 1: PRISM flow chart for article search to the system. [file 2892734.f1.pdf]
